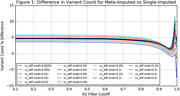# Improving Genotype Imputation of African‐derived Genetic Variants in Studies of Alzheimer's Disease

**DOI:** 10.1002/alz70855_106790

**Published:** 2025-12-25

**Authors:** Nicholas R. Wheeler, Kara L Hamilton‐Nelson, Adam C. Naj, Christiane Reitz, Scott M Williams, Giuseppe Tosto, Goldie S Byrd, Joshua O Akinyemi, Larry D Adams, Motunrayo Coker, Kazeem Akinwande, Samuel Diala, Patrice G Whitehead, Mayowa Ogunronbi, Kyle M Scott, Albertino Damasceno, Andrew F Zaman, Yared Z Zewde, Biniyam A Ayele, Allison M Caban‐Holt, David Ndetei, Anthony J Griswold, Fred Stephen Sarfo, Susan H. Blanton, Albert Akpalu, Michael L Cuccaro, Kolawole Wahab, Katalina F. McInerney, Reginald Obiako, Olusegun Baiyewu, Pedro R Mena, Njideka U Okubadejo, Mayowa O Owolabi, Izri M Martinez, Jeffery M Vance, Raj Kalaria, Adesola Ogunniyi, Farid Rajabli, Jonathan L Haines, Rufus O. Akinyemi, Margaret Pericak‐Vance, Brian W Kunkle, William S Bush, African Dementia Consortium (AfDC)

**Affiliations:** ^1^ Cleveland Institute for Computational Biology, Department of Population & Quantitative Health Sciences, School of Medicine, Case Western Reserve University, Cleveland, OH, USA; ^2^ John P. Hussman Institute for Human Genomics, University of Miami Miller School of Medicine, Miami, FL, USA; ^3^ Penn Neurodegeneration Genomics Center and Department of Biostatistics, Epidemiology, and Informatics, University of Pennsylvania, Philadelphia, PA, USA; ^4^ Gertrude H. Sergievsky Center, Taub Institute for Research on the Aging Brain, Departments of Neurology, Psychiatry, and Epidemiology, College of Physicians and Surgeons, Columbia University, New York, NY, USA; ^5^ Case Western Reserve University School of Medicine, Cleveland, OH, USA; ^6^ Wake Forest University School of Medicine, Winston‐Salem, NC, USA; ^7^ College of Medicine, University of Ibadan, Ibadan, Oyo, Nigeria; ^8^ Cell Biology and Genetics Unit, Department of Zoology, University of Ibadan, Ibadan, Nigeria; ^9^ University of Miami Miller School of Medicine, Miami, FL, USA; ^10^ Faculty of Medicine, Eduardo Mondlane University, Maputo, Mozambique; ^11^ College of Health Sciences, Addis Ababa University, Addis Ababa, Ethiopia; ^12^ Komfo Anokye Teaching Hospital, Kumasi, Ghana; ^13^ Dr. John T. Macdonald Foundation Department of Human Genetics, University of Miami Miller School of Medicine, Miami, FL, USA; ^14^ University of Ghana Medical School, Accra, Ghana; ^15^ Department of Medicine, University of Ilorin, Ilorin, Nigeria; ^16^ Department of Neurology, University of Miami Miller School of Medicine, Miami, FL, USA; ^17^ Neurology Unit, Department of Medicine, Ahmadu Bello University/Ahmadu Bello University Teaching Hospital, Zaria, Nigeria; ^18^ College of Medicine, University of Ibadan, Ibadan, Nigeria; ^19^ College of Medicine, University of Lagos, Lagos, Nigeria; ^20^ Institute for Advanced Medical Research and Training, College of Medicine, University of Ibadan, Ibadan, Oyo, Nigeria; ^21^ Newcastle University, Translational and Clinical Research Institutetitute for Ageing and Health, Newcastle UniversityNewcastle University, Newcastle upon Tyne, United Kingdom; ^22^ Institute for Advanced Medical Research and Training, College of Medicine, University of Ibadan, Ibadan, Nigeria; ^23^ Institute of Advanced Medical Research and Training, College of Medicine, University of Ibadan, Ibadan, Oyo State, Nigeria

## Abstract

**Background:**

The DAWN Alzheimer's Research Study is a multi‐site international project to recruit African‐American, Hispanic/Latino, and African participants for genomic studies of Alzheimer's Disease (AD). In addition to clinical evaluations, cognitive assessments and biomarker data collection, array‐based representative genotyping is being performed for all participants. To increase the value of these genotypic data a vastly richer dataset can be created using imputation, a process that requires a whole genome sequenced reference dataset. High quality imputation depends on having large reference datasets representative of the ancestries of the target dataset. Using inadequate reference datasets results in low imputation quality, fewer usable imputed variants and hinders downstream analysis. Given the inclusion of African‐ancestry participants (whose reference datasets are small) in the DAWN study, we examined the impact of using different strategies on the accuracy of genotype imputation.

**Method:**

Using DAWN study data generated by the Illumina Global Screening Array, we performed genotype imputation using the TopMED R3 dataset and compared these results to a meta‐imputation workflow using TopMED R3 supplemented by the Africa 6K dataset. This comparison explicitly tests the impact of increasing African ancestry in the imputation reference panel. Imputation results were assessed for chromosomes 1, 10, and 20 for total count of imputed variants, and by comparing variant counts across a range of imputation quality (R2) and variant rarity (MAF) filter criteria to identify apparent trends.

**Result:**

An additional 190,784 (0.3%) variants are captured from the meta‐imputed (64,370,296) vs the single‐imputed (64,179,512) dataset. Variant quality also improves, with an increase of ∼80,000 (5%) filter‐passing variants (R2 > 0.8) in the meta‐imputation compared to the TopMED‐only imputation results (Figure 1).

**Conclusion:**

The use of meta‐imputation to better match the genetic background of the DAWN dataset through the use of multiple imputation references significantly increases the density and quality of the resulting genotypic dataset, enabling more powerful studies of AD genetics. This demonstrates the utility of meta‐imputation for better matching the genetic background of samples when performing imputation.